# Androgenic Anabolic Steroid, Cocaine and Amphetamine Abuse and Adverse Cardiovascular Effects

**DOI:** 10.5812/ijem.8755

**Published:** 2013-10-01

**Authors:** Efren Martinez-Quintana, Beatriz Saiz-Udaeta, Natalia Marrero-Negrin, Xavier Lopez-Mérida, Fayna Rodriguez-Gonzalez, Vicente Nieto-Lago

**Affiliations:** 1 Cardiology Service, Insular-Materno Infantil University Hospital, Las Palmas de Gran Canaria, Spain; 2 Endocrinology Service, Insular-Materno Infantil University Hospital, Las Palmas de Gran Canaria, Spain; 3 Ophthalmology Service, Dr. Negrin University Hospital, Las Palmas de Gran Canaria, Spain

**Keywords:** Androgenic Anabolic Steroid, Cocaine, Amphetamine, Cardiac, Adverse Effects, Abuse

## Abstract

**Introduction:**

Anabolic-androgenic steroids (AAS), a synthetic derivate of testosterone, have become a popular drug among athletes and bodybuilders to enhance muscle mass and improve the athletic performance. Many pathological effects such as hepatic and endocrine dysfunction, behavioural changes and cardiovascular complications have been reported.

**Case Report:**

Within these ast ones, we find an increase in left ventricular muscle mass, concentric myocardial hypertrophy, left ventricular diastolic dysfunction, arterial hypertension, prothrombotic effects, changes in the concentration of cholesterol levels, particularly a reduction in HDL cholesterol concentration, myocardial infarctions in relation to endothelial dysfunction, vasospasms or thrombosis and sudden cardiac death.

**Discussion:**

We report the case of a 32-year-old patient with a history of arterial hypertension, depressive syndrome and consumption of cocaine, amphetamines and AAS who developed severe left ventricular systolic dysfunction and myocardial hypertrophy with signs of heart failure and peripheral arterial embolism.

## 1. Introduction

Anabolic-androgenic steroids (AAS) are synthetic derivates of testosterone used primarily for hormone replacement in male hypogonadism. However, in many countries, this medication is sold over-the-counter to enhance muscle mass and improve the athletic performance. Physiologically, elevations in testosterone concentrations stimulate protein synthesis resulting in improvements in muscle size, body mass and strength. AAS is by far the most detected doping substance banned by all major sporting bodies. AAS can cause many adverse effects such hepatic failure, endocrine dysfunction, behavioural changes or cardiovascular complications depending on the length and dose-dependent of drug abuse.

## 2. Case Report

A 32-year-old patient with episodes of arterial hypertension self-treated with beta blockers, depressive syndrome and frequent consumption, in adolescence and youth, of cocaine, amphetamines and AAS (750 mg of testosterone plus 750 mg nandrolone weekly in alternating cycles of 6 weeks and 3 weeks off from the age of 22) attended to the emergency department due to headache and abdominal pain in association with a hypertensive crisis (220/100 mmHg). The patient had an athletic constitution, with a weight of 109 kg and a body mass index of 33.3 kg/m2, and referred in the last months exercise intolerance attributing his current clinical symptomatology to the intake of undercooked meat (the patient referred to eat 3 kilograms of rice and 2 kilograms of meat, distributed in six meals, every day to gain muscle mass). Three days after requesting voluntary hospital discharge, the patient returned to the emergency department with intense weakness, deep sweating and severe arterial hypotension after beta blocker intake, requiring fluid and catecholamines perfusion for a few hours. Analytically, there was leukocytosis (19.5 10 3/µL) with an impairment of the renal function (creatinine of 1.7 mg/dL), an alteration of the lipid metabolism (total cholesterol of 279 mg/dL, low-density lipoprotein (LDL) of 206 mg/dL, high-density lipoprotein (HDL) of 21 mg/dL and triglyderides of 259 mg/dL) and an elevation of the liver enzymes (glutamic-oxaloacetic transaminase (GOT) of 766 u/L and glutamic-pyruvic transaminase (GPT) of 205 U/L. Basic coagulation study was normal and urine test showed positivity for methamphetamines and barbiturates. Electrocardiogram was in sinus rhythm and the echocardiogram showed severe left ventricular dysfunction, dilation, hypertrophy and increase in the ventricular mass (an ejection fraction of 20%, a diastolic diameter of 62 mm, an interventricular septum of 17 mm with a posterior wall of 15 mm thickness and a ventricular mass of 553 grams, respectively), mild right ventricular dysfunction (tricuspid annular plane systolic excursion ]TAPSE[ of 15 mm) and no significant valvular regurgitation or ventricular thrombus. Cardiac markers were within normal limits. Abdominal ultrasonography showed increased heterogeneous echogenicity of the liver without associated focal lesions. Metanephrines and catecholamines in urine were checked to rule out pheochromocytoma, as well as thyroid-stimulating hormone (TSH) and antinuclear antibodies, which were all in normal range. Serology for Coxackie B (1-6) and A9 virus, Parvovirus B19 virus, Herpes type 6 virus, Hepatitis B, C and A viruses, human immunodeficiency virus (HIV), Leptospira interrogans, Rickettsia conorii and Coxiella burnetii were negative. The patient was discharged under angiotensin-converting-enzyme inhibitors, beta blockers and anti-aldosterone treatment emphasizing the need for a radical change in the lifestyle, type of physical exercise and eating habits.

Four months after hospital admission, the patient has ceased using anabolic steroids and refers an improvement in his functional class (New York Heart Association functional class II/IV) with weight gain and a decrease in his libido. Echocardiographically, the left ventricular ejection fraction has improved to 40% and the septal thickness has decreased slightly to 15 mm in diameter showing the left ventricular apex a hyperechoic image in relation to a non-mobile thrombus. Meanwhile, analytical data shows normal serum sex-hormone-binding globulin (SHBG) concentrations, normal serum metanephrines and normetanefrines levels and normal catecholamines, metanephrines and normetanefrinas 24-hour urine concentrations. However, testosterone levels were low (0.82 ng/mL [NV 1.75-7.81]) having the external genitalia with a normal appearance.

**Figure 1.**
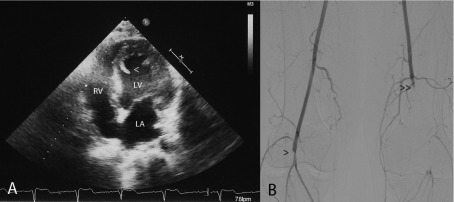
Echocardiogram and Lower Limbs Ateriography A: Apical four chamber echocardiographic view showing a thrombus, 23 mm long, attached to the ventricular septum in the left ventricular cavity (arrowhead). B: Lower limbs arteriography showing right popliteal artery (arrowhead) and left superficial femoral artery (double arrowhead) occlusion with a poor collateral circulation. LV: left ventricle, RA: right atrium, RV: right ventricle.

However 2 weeks later the patient was readmitted to the hospital due to critical ischemia of the lower limbs. Systemic heparinization associated with intravenous prostaglandin was started, presenting the patient an improvement of his symptoms with recovery of the mobility and the sensitivity. Control echocardiography showed severe global left ventricular dysfunction with a pedunculated mobile thrombus adhered to the ventricular septum (Figure 1A). Arteriography of the lower limbs showed right popliteal artery and left superficial femoral artery occlusion with a poor collateral circulation (Figure 1B). Given the improvement and little chance of surgical treatment due to the severe distal obliterations, conservative treatment and outpatient control was decided under oral anticoagulation treatment.

## 3. Discussion

Anabolic steroids have become a popular drug among athletes and are known to have a multitude of pathological effects when administered in suprapharmacological doses. Serious adverse effects include hepatic and endocrine dysfunction, behavioral changes and cardiovascular complications such as arterial hypertension, especially in those with pre-existing hypertension (1), myocardial infarction (2), myocardial hypertrophy with diastolic dysfunction (3), congestive heart failure, ventricular arrhythmias, sudden death, arterial and ventricular thrombosis (4), stroke and dyslipidemia (5). In fact, changes in the concentration of blood lipoproteins, particularly an increase in LDL and a reduction in HDL cholesterol, can lead to early atherosclerosis. However, and because of their youth, myocardial infarction in AAS consumers usually occur due to endothelial dysfunction, vasospasms or hypercoagulability (6).

Meanwhile, left ventricular hypertrophy (LVH) differential diagnosis may be considered in arterial hypertension, hypertrophic cardiomyopathy, accumulation myocardial diseases, non-compact myocardium, valvular and combined cardiac pathology, compensatory LVH in athletes and the consumption of anabolic steroids. In this context, Nieminen et al. (4) reported four patients with large doses of anabolic steroids abuse and myocardial hypertrophy, of which two patients had symptoms and signs of heart failure, and one of these two had a massive thrombosis in both the right and the left ventricles. However, after cessation of the use of anabolic steroids left ventricular and wall thickness reduced quickly and left ventricular ejection fraction increased. Endomyocardial biopsy revealed increased fibrosis in the myocardium in two of the three cases. On the contrary, Urhausen et al. (7) found, several years after discontinuation of anabolic steroid abuse, that strength athletes still showed a slight concentric LVH in comparison with anabolic androgenic steroids-free strength athletes. Similarly, D'Andrea et al. (8) in a study to investigate left ventricular dysfunction, after chronic misuse of AAS in athletes, showed that power athletes had a subclinical impairment of both systolic and diastolic myocardial functions (6), being the dysfunction associated with mean dosage and duration of AAS use. In fact, anabolic steroids consumers administer them in alternating cycles, and at doses much higher than usual, to maximize end-organ effects, to prevent gradual loss of benefits with chronic usage and to avoid detection on drug testing. In this context, the pituitary–testicular axis frequently becomes suppressed resulting in testicular atrophy, azoospermia, gynecomastia, hot flushes and fluid retention. 

Also, unfavorable cardiovascular events have been linked to both cocaine and anabolic-androgenic steroid abuse in healthy, physically active individuals (9). Cocaine can cause myocardial infarction in the context of coronary arteries spasms, enhanced myocardial oxygen demand and a procoagulant effect. Meanwhile, global ventricular dysfunction has been related to the cardiotoxic effects of the catecholamines on the heart. Similarly, the main cardiovascular manifestations of methamphetamine abuse encompass tachycardia, atrioventricular arrhythmias, myocardial ischemia and hypertension (10). 

It is also important to draw attention to the fact that drug abuse addictions and psychiatric disorders often occur at the same time having certain mental conditions such as depression, bipolar disorders, psychosis, aggression and violence, mania, suicide and symptoms of dependence and withdrawal on discontinuation associated with AAS consumption (11, 12). 

Because the over-the-counter availability and unrestrained self-medication with products containing AAS create a heightened potential for serious side effects, we should be aware of those bodybuilder patients, especially if they have a psychiatric disorder background with the consumption of other types of drugs.
